# *catena*-Poly[[[*O*,*O*′-bis­(2-methyl­phen­yl) dithio­phosphato-κ^2^ *S*,*S*]lead(II)]-μ-*O*,*O*′-bis­(2-methyl­phen­yl) dithio­phosphato-κ^3^ *S*,*S*′:*S*]

**DOI:** 10.1107/S1600536812047964

**Published:** 2012-12-08

**Authors:** Ray J. Butcher, Raju Ratnani, Sema Öztürk Yildirim, Oluwaseun Falola

**Affiliations:** a Department of Chemistry, Howard University, 525 College Street NW, Washington, DC 20059, USA; b Department of Pure & Applied Chemistry, M.D.S. University, Ajmer 305 009, India; c Department of Physics, Faculty of Sciences, Erciyes University, 38039 Kayseri, Turkey

## Abstract

In the title compound, [Pb(C_14_H_14_O_2_PS_2_)_2_]_*n*_, the metal atom is surrounded by two *O*,*O*′-bis­(2-methyl­phen­yl) dithio­phosphate ligands bonding through the S-donor atoms. Three of the Pb—S bond lengths are are close to each other at 2.7710 (18), 2.8104 (16) and 2.8205 (16) Å, while the fourth Pb—S bond is elongated at 3.0910 (18) Å and reflects the fact that this atom is involved in inter­molecular bridging to an adjacent Pb^II^ atom [Pb—S = 3.145 (2) Å]. The bond angles demonstrate that the Pb^II^ atom contains a stereochemically active lone pair with a distorted octa­hedral geometry about the Pb^II^ atom. This distortion is shown by the S—Pb—S bite angles of 73.63 (4) and 69.50 (4)°, while the remaining S—Pb—S angles range from 81.03 (5) to 143.66 (5)°. One of the benzene rings shows positional disorder over two orientations with occupancy factors of 0.747 (11) and 0.253 (11).

## Related literature

For applications of related *O*,*O*′-dialkyl derivatives of phospho­rus(V) dithio­acids, see: Lawton & Kokotailo (1969 ▶, 1972 ▶); Ito (1972 ▶); Harrison *et al.* (1988 ▶). For general and convenient methods for the preparation of dithio­phosphato salt derivatives and their metal derivatives, see: Bajia *et al.* (2009 ▶); Maheshwari *et al.* (2009 ▶); Lawton & Kokotailo (1969 ▶, 1972 ▶); Ito (1972 ▶); Harrison *et al.* (1988 ▶); Van Zyl & Fackler, (2000 ▶); Van Zyl (2010 ▶). For VSEPR theory, see: Gillespie & Nyholm (1957 ▶). For stereochemically active lone pairs in Pb^2+^ complexes, see: Davidovich *et al.* (2010 ▶); Ito & Maeda (2004 ▶); Larsson *et al.* (2004 ▶); Lawton & Kokotailo (1972 ▶). 
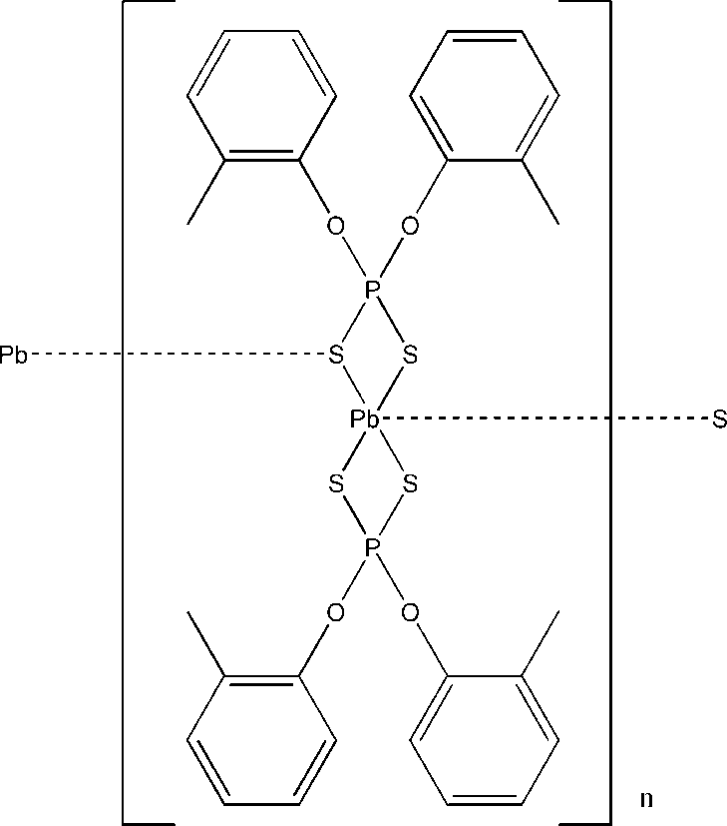


## Experimental

### 

#### Crystal data

[Pb(C_14_H_14_O_2_PS_2_)_2_]
*M* *_r_* = 825.87
Monoclinic, 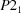

*a* = 12.0263 (6) Å
*b* = 10.7420 (4) Å
*c* = 13.0499 (8) Å
β = 112.849 (6)°
*V* = 1553.58 (15) Å^3^
*Z* = 2
Cu *K*α radiation
μ = 14.31 mm^−1^
*T* = 123 K
0.46 × 0.05 × 0.03 mm

#### Data collection

Agilent Xcalibur (Ruby, Gemini) diffractometer
Absorption correction: analytical [*CrysAlis PRO* (Agilent, 2011 ▶), using a multi-faceted crystal model (Clark & Reid, 1995 ▶)] *T*_min_ = 0.094, *T*_max_ = 0.675
10226 measured reflections
4494 independent reflections
4269 reflections with *I* > 2σ(*I*)
*R*_int_ = 0.045

#### Refinement

*R*[*F* ^2^ > 2σ(*F* ^2^)] = 0.030
*wR*(*F* ^2^) = 0.075
*S* = 1.03
4494 reflections
406 parameters
55 restraints
H-atom parameters constrained
Δρ_max_ = 1.12 e Å^−3^
Δρ_min_ = −1.15 e Å^−3^
Absolute structure: Flack (1983 ▶), 1093 Friedel pairs
Flack parameter: −0.03 (8)

### 

Data collection: *CrysAlis PRO* (Agilent, 2011 ▶); cell refinement: *CrysAlis PRO*; data reduction: *CrysAlis PRO*; program(s) used to solve structure: *SHELXS97* (Sheldrick, 2008 ▶); program(s) used to refine structure: *SHELXL97* (Sheldrick, 2008 ▶); molecular graphics: *SHELXTL* (Sheldrick, 2008 ▶); software used to prepare material for publication: *SHELXTL*.

## Supplementary Material

Crystal structure: contains datablock(s) I, global. DOI: 10.1107/S1600536812047964/bt6859sup1.cif

Structure factors: contains datablock(s) I. DOI: 10.1107/S1600536812047964/bt6859Isup2.hkl

Additional supplementary materials:  crystallographic information; 3D view; checkCIF report

## Figures and Tables

**Fig. 1.**
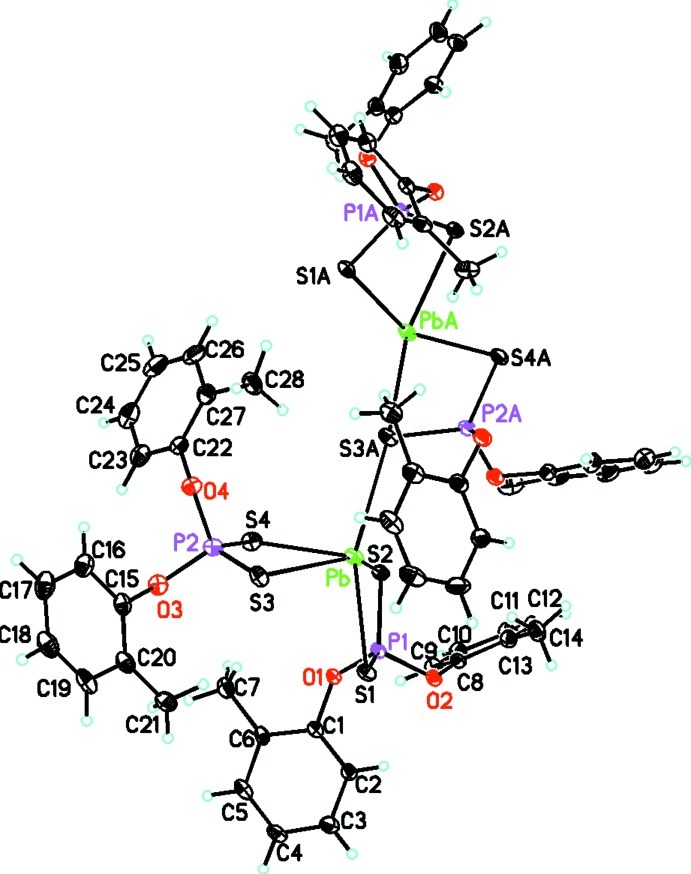
A perspective view of the title compound, showing the atom-numbering scheme. Displacement ellipsoids are drawn at the 30% probability level. Only major disordered component for the 1-methoxy-2-methyl-benzene ring is shown. Atoms labelled with suffix A were generated by the symmetry operator 2 - x, 0.5 + y, 1 - z.

**Fig. 2.**
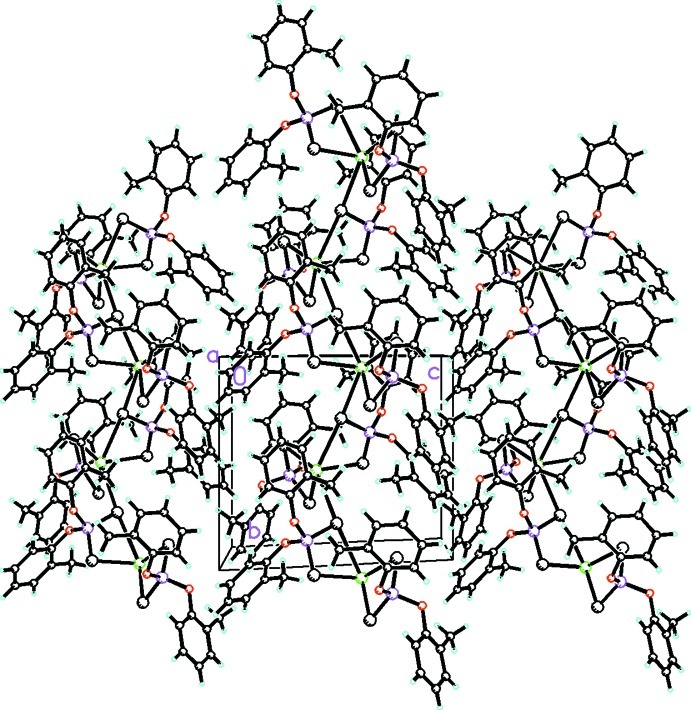
Crystal packing diagram for the title compound.

## supplementary crystallographic information

### Comment

*O*,*O*'-Dialkyl derivatives of phosphorus(V) dithioacids are characterized by wide possibilities for practical applications in various areas, namely, as flotation reagents (in the concentration of sulfide ores of nonferrous metals), fungicides, insecticides, herbicides, antioxidants, additives to lubricating oils, and technological precursors of film sulfides of transition and nontransition metals. The structural variety of metal complexes with dialkyldithiophosphates has been explained in terms of coordination chemistry by the ability of these compounds to perform different structural functions and act as bidentate terminal, bidentate bridging or combined ligands. As a result, compounds with different types of structural organization can be formed: mono-, bi-, tetra-, or polynuclear complexes. A unique alternation of the conformationally different (`chair`-`saddle`) eight-membered rings [Cd_2_S_4_P_2_] has been revealed in the chains of polynuclear cadmium(II) complexes [Cd{S(S)P(OR)_2_}_2_]_n_ (Lawton & Kokotailo, 1969; Lawton & Kokotailo, 1972; Ito, 1972; Harrison *et al.*, 1988). General and convenient methods to prepare dithiophosphato salt derivatives have been reported (Van Zyl & Fackler, 2000; Van Zyl, 2010). In view of the importance of these compounds and in continuation of our earlier work (Bajia *et al.*, 2009; Maheshwari *et al.*, 2009) we have undertaken the crystal structure determination of the title compound, and the results are presented here. Pb^2+^ complexes of these types of ligands are of particular interest because of the possibility of exhibiting stereochemically active lone pairs (Davidovich *et al.*, 2010; Ito & Maeda, 2004; Larsson *et al.*, 2004; Lawton & Kokotailo, 1972).

The X-ray study confirmed the molecular structure and atomic connectivity for (I), as illustrated in Fig. 1. The structure consists of a linear zigzag chain of molecules in the b direction composed of one Pb atom and two chelating bis(2-methylphenyl) phosphato ligands and linked by Pb—S—Pb bonds. The two bis(2-methylphenyl) phosphato ligands are coordinated through both S atoms to the metal. Three of the Pb—S bond lengths are insignificantly different at 2.7710 (18), 2.8104 (16) and 2.8205 (16) Å, while the fourth Pb—S bond is elongated at 3.0910 (18) Å and reflects the fact that this atom is involved in intermolecular bridging (symmetry code, 2 - *x*,1/2 + *y*,1 - *z*) to an adjacent Pb (intermolecular Pb—S distance, 3.145 (2) Å).

The bond angles reflect the fact that Pb contains a stereochemically active lone pair so the geometry about the Pb is best described using VSEPR theory as A*X*_5_E (Gillespie & Nyholm, 1957) and is thus distorted octahedral. The S—Pb—S bite angles are small at 73.63 (4) and 69.50 (4)° while the remaining S—Pb—S angles range from 81.03 (5) to 143.66 (5)°. Thus the relative bond distances and angles for the title compound agree with the presence of an electron lone pair in a distorted octahedral PbS_5_E (with one S as a bridging ligand) environment. Evidence for the presence of a stereochemically active electron lone pair of the lead atom has also been reported for other Pb^2+^ complexes with similar ligands (Davidovich *et al.*, 2010; Ito & Maeda, 2004; Larsson *et al.*, 2004; Lawton & Kokotailo, 1972).

In the molecule one of the 1-methoxy-2-methyl-benzene rings (O4—C22—C28) shows positional disorder over two orientations with occupancy ratio of 0.747 (11):0.253 (11).

No evidence for C—H···O or C—H···S interactions were found in the crystal.

### Experimental

Title compound was published methods (Bajia *et al.*, 2009; Maheshwari *et al.*, 2009; Lawton & Kokotailo, 1969; Lawton & Kokotailo, 1972; Ito, 1972; Harrison *et al.*, 1988). Crystals were grown by slow evaporation of a mixture of absolute ethyl alcohol (90%) and chloroform (10%) solution.

### Refinement

All H atoms were positioned geometrically and refined as riding atoms, with C—H = 0.93 (CH) and 0.96(CH_3_) Å and with *U*_iso_(H) = 1.2*U*_eq_(C). The highest residual electron density was found 0.69 Å from Pb the deepest hole 0.82 Å from Pb.

In the molecule one 1-methoxy-2-methyl-benzene ring (O4—C22—C28) shows positional disorder in a 0.747 (1):0.253 (1) ratio. The highest maximum (0.69 e/Å_3_) in the final difference map is at 1.12 Å from Pb and the deepest hole (0.82 e/Å_3_) is at -1.15 Å from Pb. Nine outliers, (-12 - 5 12), (-13 - 4 11), (-14 - 3 10), (-12 - 6 10), (-7 - 11 7), (-13 - 5 10), (0 - 8 12), (0 1 15) and (-5 - 12 6), were omitted in the final refinement.

The SIMU and DELU constraint instructions in *SHELXL97* were used for atoms O4/O4a, C22/C22*a*, C23/C23*a*, C24/C24*a*, C25/C25*a*, C26/C26*a*, C27/C27*a*,C28/C28*a* and ISOR (0.01) was used for atoms C24*a* and C26*a* in order to model the disorder properly during the refinement.

### Crystal data

**Table:**

[Pb(C_14_H_14_O_2_PS_2_)_2_]	*F*(000) = 808
*M**_r_* = 825.87	*D*_x_ = 1.765 Mg m^−^^3^
Monoclinic, *P*2_1_	Cu *K*α radiation, λ = 1.54184 Å
Hall symbol: P 2yb	Cell parameters from 4674 reflections
*a* = 12.0263 (6) Å	θ = 3.7–75.4°
*b* = 10.7420 (4) Å	µ = 14.31 mm^−^^1^
*c* = 13.0499 (8) Å	*T* = 123 K
β = 112.849 (6)°	Needle, colorless
*V* = 1553.58 (15) Å^3^	0.46 × 0.05 × 0.03 mm
*Z* = 2	

### Data collection

**Table:**

Agilent Xcalibur (Ruby, Gemini) diffractometer	4494 independent reflections
Radiation source: Enhance (Cu) X-ray Source	4269 reflections with *I* > 2σ(*I*)
Graphite monochromator	*R*_int_ = 0.045
Detector resolution: 10.5081 pixels mm^-1^	θ_max_ = 75.6°, θ_min_ = 3.7°
ω scans	*h* = −15→14
Absorption correction: analytical [*CrysAlis PRO* (Agilent, 2011), using a multi-faceted crystal model (Clark & Reid, 1995)]	*k* = −13→8
*T*_min_ = 0.094, *T*_max_ = 0.675	*l* = −14→16
10226 measured reflections	

### Refinement

**Table:**

Refinement on *F*^2^	Secondary atom site location: difference Fourier map
Least-squares matrix: full	Hydrogen site location: inferred from neighbouring sites
*R*[*F*^2^ > 2σ(*F*^2^)] = 0.030	H-atom parameters constrained
*wR*(*F*^2^) = 0.075	*w* = 1/[σ^2^(*F*_o_^2^) + (0.0329*P*)^2^ + 1.1401*P*] where *P* = (*F*_o_^2^ + 2*F*_c_^2^)/3
*S* = 1.03	(Δ/σ)_max_ = 0.007
4494 reflections	Δρ_max_ = 1.12 e Å^−^^3^
406 parameters	Δρ_min_ = −1.15 e Å^−^^3^
55 restraints	Absolute structure: Flack (1983), 1093 Friedel pairs
Primary atom site location: structure-invariant direct methods	Flack parameter: −0.03 (8)

### Special details

**Table:**

Geometry. All e.s.d.'s (except the e.s.d. in the dihedral angle between two l.s. planes) are estimated using the full covariance matrix. The cell e.s.d.'s are taken into account individually in the estimation of e.s.d.'s in distances, angles and torsion angles; correlations between e.s.d.'s in cell parameters are only used when they are defined by crystal symmetry. An approximate (isotropic) treatment of cell e.s.d.'s is used for estimating e.s.d.'s involving l.s. planes.
Refinement. Refinement of *F*^2^ against ALL reflections. The weighted *R*-factor *wR* and goodness of fit *S* are based on *F*^2^, conventional *R*-factors *R* are based on *F*, with *F* set to zero for negative *F*^2^. The threshold expression of *F*^2^ > σ(*F*^2^) is used only for calculating *R*-factors(gt) *etc*. and is not relevant to the choice of reflections for refinement. *R*-factors based on *F*^2^ are statistically about twice as large as those based on *F*, and *R*- factors based on ALL data will be even larger.

### Fractional atomic coordinates and isotropic or equivalent isotropic displacement parameters (Å^2^)

**Table:**

	*x*	*y*	*z*	*U*_iso_*/*U*_eq_	Occ. (<1)
P1	0.58111 (14)	0.59159 (15)	0.27999 (13)	0.0313 (3)	
P2	0.94672 (15)	0.36924 (17)	0.63559 (15)	0.0397 (4)	
S1	0.65999 (14)	0.43767 (15)	0.25837 (14)	0.0372 (3)	
S2	0.68892 (13)	0.72496 (15)	0.37035 (14)	0.0360 (3)	
S3	0.97595 (15)	0.29732 (17)	0.50949 (16)	0.0426 (4)	
S4	0.84998 (14)	0.52418 (15)	0.60457 (14)	0.0428 (4)	
Pb	0.874757 (17)	0.55165 (2)	0.403361 (19)	0.03788 (6)	
O1	0.4862 (3)	0.5601 (6)	0.3355 (3)	0.0329 (9)	
O2	0.5005 (4)	0.6443 (4)	0.1582 (4)	0.0354 (9)	
O3	0.8792 (4)	0.2724 (5)	0.6864 (4)	0.0452 (12)	
C1	0.4143 (5)	0.4517 (6)	0.3029 (5)	0.0334 (13)	
C2	0.3246 (6)	0.4438 (7)	0.1981 (6)	0.0379 (14)	
H2A	0.3119	0.5086	0.1476	0.045*	
C3	0.2528 (7)	0.3365 (8)	0.1688 (7)	0.0470 (17)	
H3A	0.1930	0.3288	0.0978	0.056*	
C4	0.2710 (7)	0.2427 (7)	0.2451 (7)	0.0483 (17)	
H4A	0.2230	0.1717	0.2261	0.058*	
C5	0.3608 (7)	0.2538 (7)	0.3502 (7)	0.0453 (16)	
H5A	0.3726	0.1893	0.4008	0.054*	
C6	0.4350 (5)	0.3602 (6)	0.3828 (5)	0.0350 (13)	
C7	0.5306 (6)	0.3735 (8)	0.4978 (7)	0.0502 (19)	
H7A	0.5198	0.4510	0.5295	0.075*	
H7B	0.6088	0.3722	0.4941	0.075*	
H7C	0.5244	0.3059	0.5434	0.075*	
C8	0.4551 (6)	0.7671 (6)	0.1443 (5)	0.0359 (13)	
C9	0.3744 (6)	0.8024 (8)	0.1895 (6)	0.0424 (15)	
H9A	0.3508	0.7464	0.2316	0.051*	
C10	0.3283 (7)	0.9222 (8)	0.1720 (7)	0.0490 (17)	
H10A	0.2754	0.9479	0.2042	0.059*	
C11	0.3614 (8)	1.0035 (8)	0.1062 (7)	0.0534 (19)	
H11A	0.3286	1.0832	0.0918	0.064*	
C12	0.4437 (9)	0.9654 (8)	0.0621 (6)	0.054 (2)	
H12A	0.4653	1.0207	0.0181	0.064*	
C13	0.4950 (7)	0.8473 (7)	0.0814 (6)	0.0407 (15)	
C14	0.5853 (8)	0.8076 (8)	0.0345 (7)	0.0540 (19)	
H14A	0.6487	0.7610	0.0895	0.081*	
H14B	0.6188	0.8798	0.0139	0.081*	
H14C	0.5461	0.7565	−0.0299	0.081*	
C15	0.9162 (7)	0.1475 (8)	0.7050 (7)	0.0428 (17)	
C16	1.0210 (8)	0.1178 (10)	0.7962 (7)	0.051 (2)	
H16A	1.0669	0.1801	0.8430	0.061*	
C17	1.0567 (9)	−0.0087 (11)	0.8169 (9)	0.065 (3)	
H17A	1.1253	−0.0315	0.8779	0.078*	
C18	0.9857 (8)	−0.0971 (9)	0.7428 (9)	0.061 (2)	
H18A	1.0086	−0.1803	0.7544	0.073*	
C19	0.8856 (8)	−0.0673 (8)	0.6553 (8)	0.055 (2)	
H19A	0.8409	−0.1305	0.6088	0.065*	
C20	0.8456 (5)	0.0560 (12)	0.6314 (6)	0.0472 (15)	
C21	0.7325 (7)	0.0918 (8)	0.5346 (7)	0.0509 (19)	
H21A	0.7496	0.1594	0.4949	0.076*	
H21B	0.6720	0.1171	0.5613	0.076*	
H21C	0.7036	0.0217	0.4858	0.076*	
O4	1.0800 (7)	0.3917 (8)	0.7289 (7)	0.043 (2)	0.747 (11)
C22	1.0991 (7)	0.4823 (6)	0.8088 (6)	0.040 (3)	0.747 (11)
C23	1.0368 (7)	0.4817 (8)	0.8790 (7)	0.058 (3)	0.747 (11)
H23	0.9802	0.4199	0.8721	0.069*	0.747 (11)
C24	1.0591 (9)	0.5736 (10)	0.9595 (6)	0.077 (6)	0.747 (11)
H24	1.0175	0.5733	1.0065	0.093*	0.747 (11)
C25	1.1437 (10)	0.6661 (8)	0.9699 (6)	0.080 (5)	0.747 (11)
H25	1.1586	0.7276	1.0238	0.096*	0.747 (11)
C26	1.2060 (8)	0.6666 (7)	0.8997 (8)	0.071 (5)	0.747 (11)
H26	1.2625	0.7285	0.9066	0.086*	0.747 (11)
C27	1.1836 (7)	0.5747 (7)	0.8192 (7)	0.058 (3)	0.747 (11)
C28	1.2475 (11)	0.5753 (14)	0.7405 (11)	0.064 (4)	0.747 (11)
H28A	1.3050	0.6422	0.7596	0.096*	0.747 (11)
H28B	1.1897	0.5867	0.6658	0.096*	0.747 (11)
H28C	1.2887	0.4976	0.7458	0.096*	0.747 (11)
O4A	1.0570 (17)	0.374 (2)	0.762 (2)	0.044 (6)	0.253 (11)
C22A	1.1269 (15)	0.4788 (17)	0.7926 (16)	0.054 (11)	0.253 (11)
C23A	1.2071 (17)	0.5125 (19)	0.7443 (16)	0.048 (8)	0.253 (11)
H23A	1.2118	0.4653	0.6863	0.057*	0.253 (11)
C24A	1.2802 (16)	0.617 (2)	0.7825 (18)	0.045 (8)	0.253 (11)
H24A	1.3339	0.6391	0.7502	0.055*	0.253 (11)
C25A	1.2731 (19)	0.6870 (18)	0.8691 (18)	0.060 (10)	0.253 (11)
H25A	1.3221	0.7567	0.8947	0.072*	0.253 (11)
C26A	1.193 (2)	0.653 (2)	0.9175 (16)	0.045 (8)	0.253 (11)
H26A	1.1882	0.7005	0.9755	0.054*	0.253 (11)
C27A	1.1198 (16)	0.549 (2)	0.8793 (16)	0.053 (7)	0.253 (11)
C28A	1.030 (3)	0.511 (6)	0.927 (3)	0.09 (2)	0.253 (11)
H28D	1.0442	0.4262	0.9514	0.134*	0.253 (11)
H28E	0.9498	0.5189	0.8710	0.134*	0.253 (11)
H28F	1.0381	0.5638	0.9889	0.134*	0.253 (11)

### Atomic displacement parameters (Å^2^)

**Table:**

	*U*^11^	*U*^22^	*U*^33^	*U*^12^	*U*^13^	*U*^23^
P1	0.0341 (6)	0.0230 (7)	0.0404 (7)	0.0005 (5)	0.0186 (6)	0.0006 (5)
P2	0.0325 (7)	0.0354 (9)	0.0492 (9)	0.0053 (7)	0.0139 (6)	−0.0042 (7)
S1	0.0395 (7)	0.0256 (8)	0.0518 (8)	0.0014 (6)	0.0234 (6)	−0.0039 (6)
S2	0.0345 (7)	0.0259 (7)	0.0480 (8)	−0.0006 (6)	0.0163 (6)	−0.0032 (6)
S3	0.0477 (10)	0.0303 (8)	0.0595 (10)	0.0064 (8)	0.0314 (8)	0.0042 (7)
S4	0.0401 (7)	0.0366 (12)	0.0516 (8)	0.0103 (6)	0.0178 (6)	−0.0056 (6)
Pb	0.03314 (9)	0.02947 (11)	0.05628 (12)	−0.00011 (13)	0.02308 (8)	−0.00157 (14)
O1	0.0358 (16)	0.028 (2)	0.0381 (17)	−0.001 (2)	0.0180 (14)	0.001 (2)
O2	0.045 (2)	0.024 (2)	0.041 (2)	−0.0006 (18)	0.0196 (19)	0.0013 (17)
O3	0.042 (2)	0.047 (3)	0.051 (3)	0.010 (2)	0.024 (2)	0.002 (2)
C1	0.036 (3)	0.029 (3)	0.043 (3)	0.002 (2)	0.022 (2)	−0.003 (2)
C2	0.041 (3)	0.036 (4)	0.042 (3)	−0.004 (3)	0.021 (3)	0.000 (3)
C3	0.047 (4)	0.044 (4)	0.055 (4)	−0.008 (3)	0.025 (3)	−0.009 (3)
C4	0.058 (4)	0.031 (4)	0.067 (4)	−0.012 (3)	0.037 (4)	−0.010 (3)
C5	0.049 (3)	0.031 (4)	0.069 (4)	0.003 (3)	0.038 (3)	0.008 (3)
C6	0.035 (3)	0.033 (3)	0.045 (3)	0.007 (3)	0.024 (2)	0.010 (3)
C7	0.036 (3)	0.054 (5)	0.061 (4)	0.006 (3)	0.020 (3)	0.022 (4)
C8	0.040 (3)	0.028 (3)	0.038 (3)	−0.003 (3)	0.013 (2)	0.001 (2)
C9	0.042 (3)	0.041 (4)	0.046 (3)	0.004 (3)	0.019 (3)	0.002 (3)
C10	0.055 (4)	0.041 (4)	0.051 (4)	0.015 (3)	0.020 (3)	0.006 (3)
C11	0.075 (5)	0.040 (4)	0.052 (4)	0.015 (4)	0.032 (4)	0.006 (3)
C12	0.083 (5)	0.039 (4)	0.044 (4)	0.006 (4)	0.030 (4)	0.012 (3)
C13	0.054 (4)	0.028 (3)	0.042 (3)	0.003 (3)	0.021 (3)	−0.001 (3)
C14	0.079 (5)	0.038 (4)	0.058 (4)	−0.004 (4)	0.040 (4)	−0.001 (3)
C15	0.047 (4)	0.044 (4)	0.050 (4)	0.009 (3)	0.032 (3)	0.008 (3)
C16	0.050 (4)	0.062 (6)	0.045 (4)	0.012 (4)	0.024 (3)	0.006 (4)
C17	0.057 (5)	0.079 (7)	0.062 (5)	0.029 (5)	0.027 (4)	0.027 (5)
C18	0.059 (5)	0.044 (5)	0.095 (7)	0.012 (4)	0.046 (5)	0.019 (4)
C19	0.069 (5)	0.035 (4)	0.084 (6)	0.002 (4)	0.055 (5)	0.007 (4)
C20	0.044 (3)	0.047 (4)	0.063 (3)	0.001 (5)	0.034 (3)	0.010 (5)
C21	0.043 (3)	0.047 (4)	0.067 (5)	−0.003 (3)	0.027 (3)	0.001 (3)
O4	0.033 (4)	0.047 (4)	0.049 (4)	0.004 (3)	0.018 (3)	−0.007 (3)
C22	0.046 (5)	0.036 (6)	0.034 (5)	0.000 (4)	0.010 (4)	−0.007 (4)
C23	0.067 (7)	0.068 (8)	0.040 (6)	0.013 (6)	0.022 (5)	0.001 (5)
C24	0.087 (10)	0.098 (16)	0.037 (6)	0.031 (9)	0.012 (6)	−0.004 (7)
C25	0.087 (10)	0.064 (9)	0.052 (7)	0.012 (8)	−0.013 (7)	−0.013 (6)
C26	0.073 (9)	0.046 (7)	0.058 (8)	0.003 (7)	−0.015 (7)	−0.004 (6)
C27	0.050 (5)	0.054 (9)	0.055 (6)	0.001 (5)	0.003 (4)	0.017 (5)
C28	0.042 (6)	0.060 (10)	0.082 (8)	−0.012 (6)	0.014 (6)	0.020 (7)
O4A	0.014 (8)	0.058 (14)	0.056 (14)	0.003 (8)	0.010 (8)	−0.007 (11)
C22A	0.044 (17)	0.05 (2)	0.047 (18)	0.011 (15)	−0.003 (15)	0.016 (15)
C23A	0.028 (13)	0.044 (19)	0.067 (19)	−0.008 (12)	0.014 (13)	−0.015 (14)
C24A	0.039 (10)	0.044 (11)	0.051 (10)	−0.010 (8)	0.015 (8)	0.005 (8)
C25A	0.047 (17)	0.042 (18)	0.06 (2)	−0.017 (15)	−0.008 (15)	0.011 (15)
C26A	0.050 (11)	0.040 (11)	0.033 (10)	−0.003 (8)	0.005 (8)	−0.001 (8)
C27A	0.033 (10)	0.060 (16)	0.072 (15)	0.016 (18)	0.025 (10)	0.03 (2)
C28A	0.039 (15)	0.19 (8)	0.04 (2)	−0.01 (3)	0.015 (16)	−0.02 (3)

### Geometric parameters (Å, º)

**Table:**

P1—O1	1.608 (4)	C15—C16	1.394 (11)
P1—O2	1.609 (5)	C15—C20	1.406 (14)
P1—S1	1.980 (2)	C16—C17	1.419 (13)
P1—S2	1.984 (2)	C16—H16A	0.9300
P2—O3	1.612 (6)	C17—C18	1.388 (15)
P2—O4	1.611 (8)	C17—H17A	0.9300
P2—O4A	1.67 (2)	C18—C19	1.337 (14)
P2—S3	1.969 (3)	C18—H18A	0.9300
P2—S4	1.980 (2)	C19—C20	1.403 (15)
S1—Pb	2.8205 (16)	C19—H19A	0.9300
S2—Pb	2.8104 (16)	C20—C21	1.503 (10)
S3—Pb	3.0910 (18)	C21—H21A	0.9600
S4—Pb	2.7710 (18)	C21—H21B	0.9600
O1—C1	1.414 (8)	C21—H21C	0.9600
O2—C8	1.413 (8)	O4—C22	1.379 (9)
O3—C15	1.404 (10)	C22—C23	1.3900
C1—C2	1.376 (9)	C22—C27	1.3900
C1—C6	1.384 (9)	C23—C24	1.3900
C2—C3	1.402 (10)	C23—H23	0.9300
C2—H2A	0.9300	C24—C25	1.3900
C3—C4	1.373 (12)	C24—H24	0.9300
C3—H3A	0.9300	C25—C26	1.3900
C4—C5	1.382 (12)	C25—H25	0.9300
C4—H4A	0.9300	C26—C27	1.3900
C5—C6	1.410 (10)	C26—H26	0.9300
C5—H5A	0.9300	C27—C28	1.502 (14)
C6—C7	1.501 (10)	C28—H28A	0.9600
C7—H7A	0.9600	C28—H28B	0.9600
C7—H7B	0.9600	C28—H28C	0.9600
C7—H7C	0.9600	O4A—C22A	1.371 (16)
C8—C9	1.370 (10)	C22A—C23A	1.3900
C8—C13	1.397 (10)	C22A—C27A	1.3900
C9—C10	1.384 (11)	C23A—C24A	1.3900
C9—H9A	0.9300	C23A—H23A	0.9300
C10—C11	1.387 (12)	C24A—C25A	1.3900
C10—H10A	0.9300	C24A—H24A	0.9300
C11—C12	1.387 (12)	C25A—C26A	1.3900
C11—H11A	0.9300	C25A—H25A	0.9300
C12—C13	1.391 (11)	C26A—C27A	1.3900
C12—H12A	0.9300	C26A—H26A	0.9300
C13—C14	1.500 (11)	C27A—C28A	1.50 (2)
C14—H14A	0.9600	C28A—H28D	0.9600
C14—H14B	0.9600	C28A—H28E	0.9600
C14—H14C	0.9600	C28A—H28F	0.9600
			
O1—P1—O2	104.9 (2)	H14A—C14—H14B	109.5
O1—P1—S1	110.6 (2)	C13—C14—H14C	109.5
O2—P1—S1	106.93 (19)	H14A—C14—H14C	109.5
O1—P1—S2	107.4 (2)	H14B—C14—H14C	109.5
O2—P1—S2	109.71 (19)	C16—C15—O3	119.2 (8)
S1—P1—S2	116.68 (10)	C16—C15—C20	122.0 (9)
O3—P2—O4	107.3 (4)	O3—C15—C20	118.9 (7)
O3—P2—O4A	87.0 (8)	C15—C16—C17	119.2 (9)
O4—P2—O4A	21.9 (7)	C15—C16—H16A	120.4
O3—P2—S3	111.9 (2)	C17—C16—H16A	120.4
O4—P2—S3	104.0 (3)	C18—C17—C16	117.7 (9)
O4A—P2—S3	120.3 (8)	C18—C17—H17A	121.2
O3—P2—S4	106.1 (2)	C16—C17—H17A	121.2
O4—P2—S4	111.2 (3)	C19—C18—C17	122.5 (9)
O4A—P2—S4	110.9 (8)	C19—C18—H18A	118.8
S3—P2—S4	116.12 (12)	C17—C18—H18A	118.8
P1—S1—Pb	84.63 (7)	C18—C19—C20	122.3 (9)
P1—S2—Pb	84.84 (7)	C18—C19—H19A	118.9
P2—S3—Pb	81.99 (8)	C20—C19—H19A	118.9
P2—S4—Pb	90.71 (8)	C19—C20—C15	116.4 (8)
S4—Pb—S2	81.03 (5)	C19—C20—C21	123.3 (9)
S4—Pb—S1	100.49 (5)	C15—C20—C21	120.3 (10)
S2—Pb—S1	73.63 (4)	C20—C21—H21A	109.5
S4—Pb—S3	69.50 (4)	C20—C21—H21B	109.5
S2—Pb—S3	143.66 (5)	H21A—C21—H21B	109.5
S1—Pb—S3	90.89 (5)	C20—C21—H21C	109.5
C1—O1—P1	119.7 (4)	H21A—C21—H21C	109.5
C8—O2—P1	120.7 (4)	H21B—C21—H21C	109.5
C15—O3—P2	120.7 (5)	C22—O4—P2	120.1 (6)
C2—C1—C6	123.2 (6)	O4—C22—C23	121.4 (6)
C2—C1—O1	120.1 (6)	O4—C22—C27	118.6 (6)
C6—C1—O1	116.6 (6)	C23—C22—C27	120.0
C1—C2—C3	118.9 (7)	C22—C23—C24	120.0
C1—C2—H2A	120.5	C22—C23—H23	120.0
C3—C2—H2A	120.5	C24—C23—H23	120.0
C4—C3—C2	119.9 (7)	C25—C24—C23	120.0
C4—C3—H3A	120.1	C25—C24—H24	120.0
C2—C3—H3A	120.1	C23—C24—H24	120.0
C3—C4—C5	119.9 (7)	C26—C25—C24	120.0
C3—C4—H4A	120.0	C26—C25—H25	120.0
C5—C4—H4A	120.0	C24—C25—H25	120.0
C4—C5—C6	121.9 (7)	C27—C26—C25	120.0
C4—C5—H5A	119.0	C27—C26—H26	120.0
C6—C5—H5A	119.0	C25—C26—H26	120.0
C1—C6—C5	116.1 (6)	C26—C27—C22	120.0
C1—C6—C7	122.0 (6)	C26—C27—C28	120.9 (8)
C5—C6—C7	121.9 (6)	C22—C27—C28	119.1 (8)
C6—C7—H7A	109.5	C22A—O4A—P2	118.4 (18)
C6—C7—H7B	109.5	O4A—C22A—C23A	122.3 (15)
H7A—C7—H7B	109.5	O4A—C22A—C27A	117.6 (15)
C6—C7—H7C	109.5	C23A—C22A—C27A	120.0
H7A—C7—H7C	109.5	C24A—C23A—C22A	120.0
H7B—C7—H7C	109.5	C24A—C23A—H23A	120.0
C9—C8—C13	123.0 (7)	C22A—C23A—H23A	120.0
C9—C8—O2	120.4 (6)	C25A—C24A—C23A	120.0
C13—C8—O2	116.6 (6)	C25A—C24A—H24A	120.0
C8—C9—C10	119.4 (7)	C23A—C24A—H24A	120.0
C8—C9—H9A	120.3	C24A—C25A—C26A	120.0
C10—C9—H9A	120.3	C24A—C25A—H25A	120.0
C9—C10—C11	119.7 (8)	C26A—C25A—H25A	120.0
C9—C10—H10A	120.2	C27A—C26A—C25A	120.0
C11—C10—H10A	120.2	C27A—C26A—H26A	120.0
C12—C11—C10	119.6 (8)	C25A—C26A—H26A	120.0
C12—C11—H11A	120.2	C26A—C27A—C22A	120.0
C10—C11—H11A	120.2	C26A—C27A—C28A	122 (2)
C11—C12—C13	122.1 (8)	C22A—C27A—C28A	118 (2)
C11—C12—H12A	118.9	C27A—C28A—H28D	109.5
C13—C12—H12A	118.9	C27A—C28A—H28E	109.5
C12—C13—C8	116.1 (7)	H28D—C28A—H28E	109.5
C12—C13—C14	121.7 (7)	C27A—C28A—H28F	109.5
C8—C13—C14	122.2 (7)	H28D—C28A—H28F	109.5
C13—C14—H14A	109.5	H28E—C28A—H28F	109.5
C13—C14—H14B	109.5		
			
O1—P1—S1—Pb	−118.69 (17)	C10—C11—C12—C13	0.0 (14)
O2—P1—S1—Pb	127.58 (19)	C11—C12—C13—C8	2.5 (12)
S2—P1—S1—Pb	4.39 (10)	C11—C12—C13—C14	−179.2 (8)
O1—P1—S2—Pb	120.3 (2)	C9—C8—C13—C12	−2.9 (11)
O2—P1—S2—Pb	−126.2 (2)	O2—C8—C13—C12	175.9 (6)
S1—P1—S2—Pb	−4.41 (11)	C9—C8—C13—C14	178.8 (7)
O3—P2—S3—Pb	133.5 (2)	O2—C8—C13—C14	−2.4 (10)
O4—P2—S3—Pb	−110.9 (4)	P2—O3—C15—C16	75.9 (8)
O4A—P2—S3—Pb	−126.8 (9)	P2—O3—C15—C20	−104.6 (7)
S4—P2—S3—Pb	11.61 (11)	O3—C15—C16—C17	178.3 (9)
O3—P2—S4—Pb	−137.8 (2)	C20—C15—C16—C17	−1.1 (13)
O4—P2—S4—Pb	105.8 (3)	C15—C16—C17—C18	1.3 (15)
O4A—P2—S4—Pb	129.3 (8)	C16—C17—C18—C19	−1.1 (16)
S3—P2—S4—Pb	−12.84 (12)	C17—C18—C19—C20	0.7 (14)
P2—S4—Pb—S2	166.17 (8)	C18—C19—C20—C15	−0.4 (11)
P2—S4—Pb—S1	94.80 (8)	C18—C19—C20—C21	−179.1 (8)
P2—S4—Pb—S3	7.80 (7)	C16—C15—C20—C19	0.7 (10)
P1—S2—Pb—S4	−100.91 (7)	O3—C15—C20—C19	−178.7 (7)
P1—S2—Pb—S1	2.88 (7)	C16—C15—C20—C21	179.4 (7)
P1—S2—Pb—S3	−65.29 (11)	O3—C15—C20—C21	−0.1 (10)
P1—S1—Pb—S4	74.43 (7)	O3—P2—O4—C22	−87.1 (8)
P1—S1—Pb—S2	−2.89 (7)	O4A—P2—O4—C22	−65 (2)
P1—S1—Pb—S3	143.73 (7)	S3—P2—O4—C22	154.3 (7)
P2—S3—Pb—S4	−7.92 (7)	S4—P2—O4—C22	28.5 (8)
P2—S3—Pb—S2	−45.83 (12)	P2—O4—C22—C23	57.5 (9)
P2—S3—Pb—S1	−108.79 (8)	P2—O4—C22—C27	−123.2 (6)
O2—P1—O1—C1	76.3 (5)	O4—C22—C23—C24	179.3 (8)
S1—P1—O1—C1	−38.7 (5)	C27—C22—C23—C24	0.0
S2—P1—O1—C1	−167.0 (4)	C22—C23—C24—C25	0.0
O1—P1—O2—C8	77.3 (5)	C23—C24—C25—C26	0.0
S1—P1—O2—C8	−165.2 (4)	C24—C25—C26—C27	0.0
S2—P1—O2—C8	−37.8 (5)	C25—C26—C27—C22	0.0
O4—P2—O3—C15	−68.9 (6)	C25—C26—C27—C28	178.4 (8)
O4A—P2—O3—C15	−77.0 (10)	O4—C22—C27—C26	−179.3 (7)
S3—P2—O3—C15	44.6 (6)	C23—C22—C27—C26	0.0
S4—P2—O3—C15	172.1 (5)	O4—C22—C27—C28	2.3 (9)
P1—O1—C1—C2	−69.4 (7)	C23—C22—C27—C28	−178.4 (8)
P1—O1—C1—C6	114.0 (5)	O3—P2—O4A—C22A	−153.3 (16)
C6—C1—C2—C3	−2.2 (10)	O4—P2—O4A—C22A	47.8 (19)
O1—C1—C2—C3	−178.5 (6)	S3—P2—O4A—C22A	93.1 (16)
C1—C2—C3—C4	1.4 (11)	S4—P2—O4A—C22A	−47.2 (18)
C2—C3—C4—C5	−0.6 (12)	P2—O4A—C22A—C23A	−69 (2)
C3—C4—C5—C6	0.6 (12)	P2—O4A—C22A—C27A	114.4 (17)
C2—C1—C6—C5	2.0 (9)	O4A—C22A—C23A—C24A	−177 (2)
O1—C1—C6—C5	178.5 (5)	C27A—C22A—C23A—C24A	0.0
C2—C1—C6—C7	−177.7 (6)	C22A—C23A—C24A—C25A	0.0
O1—C1—C6—C7	−1.2 (9)	C23A—C24A—C25A—C26A	0.0
C4—C5—C6—C1	−1.2 (10)	C24A—C25A—C26A—C27A	0.0
C4—C5—C6—C7	178.6 (7)	C25A—C26A—C27A—C22A	0.0
P1—O2—C8—C9	−63.7 (8)	C25A—C26A—C27A—C28A	−179 (3)
P1—O2—C8—C13	117.6 (6)	O4A—C22A—C27A—C26A	177 (2)
C13—C8—C9—C10	0.7 (11)	C23A—C22A—C27A—C26A	0.0
O2—C8—C9—C10	−178.0 (6)	O4A—C22A—C27A—C28A	−5 (3)
C8—C9—C10—C11	2.0 (12)	C23A—C22A—C27A—C28A	179 (3)
C9—C10—C11—C12	−2.3 (13)		
